# YB-1 Expression Is Associated with Lymph Node Metastasis and Drug Resistance to Adriamycin in Breast Cancer

**DOI:** 10.1155/2023/4667089

**Published:** 2023-02-04

**Authors:** Hanzhi Dong, Yan Jian, Tenghua Yu, Meijian Wang, Wenfeng Zhang, Zhiqiang Peng

**Affiliations:** ^1^Department of Medical Oncology, Jiangxi Cancer Hospital, The Second Affiliated Hospital of Nanchang Medical College, Jiangxi Clinical Research Center for Cancer, Nanchang 330029, China; ^2^Department of Breast Surgery, Jiangxi Cancer Hospital, The Second Affiliated Hospital of Nanchang Medical College, Jiangxi Clinical Research Center for Cancer, Nanchang 330029, China; ^3^Department of Infectious Diseases, The First Affiliated Hospital, Nanchang University, Nanchang, 330006 Jiangxi, China; ^4^Department of Lymphohematology, Jiangxi Cancer Hospital, The Second Affiliated Hospital of Nanchang Medical College, Jiangxi Clinical Research Center for Cancer, Nanchang 330029, China

## Abstract

**Background:**

Breast cancer (BC) is the most common malignant tumor among females. Although there are multiple treatments for breast cancer, many patients still face the dilemma of drug resistance after multiline treatment. It would be greatly helpful for clinical work to identify additional and improved prognostic predictors. Y-box binding protein-1 (YB-1) is a member of the cold shock protein family, and patients with overexpression of YB-1 have a worse prognosis.

**Methods:**

This study collected 48 specimens from 48 patients with breast cancer and analyzed the clinicopathological characteristics of the patients. Immunohistochemistry, immunofluorescence, cell viability analysis, tumor spheroid formation and cell morphology, cell invasion, cycle analysis, qRT-PCR, Western blot, and tumorigenicity in BALB/c nude mice were performed to verify the results.

**Results:**

We found that patients with overexpression of YB-1 were related to lymph node metastasis and the patients' age tended to be young. Because of the short follow-up time, a survival analysis could not be performed. Based on the results of in vitro and in vivo experiments, this study indicated that breast cancer cells with overexpression of YB-1 had stronger proliferation, migration, and invasion abilities than cells with low expression of YB-1. Compared with cells with low expression of YB-1, the proliferation, migration, and invasion abilities of YB-1 overexpressed cells were not significantly affected by adriamycin.

**Conclusion:**

This suggested that breast cancer cells with overexpression of YB-1 were resistant to adriamycin. Therefore, YB-1 is associated with lymph node metastasis of breast cancer cell. YB-1 could be a prognostic, predictive factor and a novel therapeutic target of BC.

## 1. Introduction

Breast cancer (BC) is one of the most common malignancies in women, with 1.3 million cases diagnosed and 400,000 deaths worldwide each year [1, 2]. The etiologies of BC are very complex, and its onset and therapeutic effect are related to a variety of factors, such as estrogen receptor (ER), progesterone receptor (PR), human epidermal growth factor receptor 2 (HER-2), BRCA, and hormone levels [3, 4]. Studies have shown that breast cancers with triple-negative and HER-2 positive are more prone to brain metastasis. Ferreira et al. [5] found that patients with serum positive for Y-box binding protein 1 (YB-1) were more likely to have bone metastasis. However, little studies have been conducted on the relationship between tissue levels of YB-1 and prognosis of patients with stage II and III breast cancer. Anthracyclines are one of the main class of drugs used to treat breast cancer, but once the cancer cells gain resistance to anthracyclines, the treatment options are limited. A better understanding of drug resistance of BC cells is crucial to develop effective breast cancer treatments.

YB-1 is a member of the cold shock protein superfamily, and it exists widely in a variety of tissues, including both normal and tumor tissues. YB-1 has already been reported to be associated with tumor growth, proliferation, invasion, and metastasis [6]. YB-1 is a translation factor, which could act as an effector of multiple downstream signaling pathways. The expression of YB-1 is closely related to the prognosis of malignant tumors. Thus, YB-1 could be used as a prognostic factor. Its phosphorylation and nuclear localization are the key process in drug-resistant tumor cells. In addition, YB-1 is involved in the regulation of drug resistance-related genes, which may disrupt the function of drug-resistant genes, such as ATP binding cassette subfamily B member 1 (ABCB1), major vault protein (MVP)/LDL receptor-related protein 1 (LRP), proliferating cell nuclear antigen (PCNA), MYC protooncogene, BHLH transcription factor (MYC), DNA topoisomerase II alpha (TOP2A), and androgen receptor (AR) [7]. In recent years, there have been some preclinical studies [8] exploring potential target genes of YB-1. YB-1 may become a potential treatment target for antitumor drug resistance or drug resistance reversal in the future. YB-1 has promising research prospects and important clinical value.

YB-1 is frequently expressed in breast cancer cells. However, whether the level of YB-1 affects the efficacy of anthracyclines is still unknown. Therefore, in the present study, we performed a retrospective analysis of the prognostic value of YB-1 in breast cancer tissues and assessed the effects of YB-1 expression on sensitivity to anthracyclines.

## 2. Materials and Methods

### 2.1. Patients and Samples

Fifty breast cancer tissue samples were collected from patients who underwent surgery at Jiangxi Cancer Hospital from January 2016 to December 2018. The pathological diagnoses were all invasive ductal carcinomas. Additionally, the corresponding normal tissue adjacent to the carcinoma was also collected. All patients received adjuvant chemotherapy (anthracycline-based chemotherapy regimen). The mean follow-up time was 35.6 months (range: 8–49 months). Two patients were lost to follow-up. As a result, the clinical data were from 48 patients. This study was approved by the Ethics Committee of Jiangxi Cancer Hospital (No. 2021ky021).

### 2.2. Immunohistochemistry

Paraffin-embedded specimens were cut into 4 *μ*m thick slices and baked on a sheet in a constant temperature oven. Then, they were deparaffinized and rehydrated and antigen retrieval was performed. The slides were incubated with a 1 : 200 dilution of polyclonal mouse YB-1 antibody (Abcam, Item No. Ab219070) for 1 h at room temperature. Following incubation with MaxVision rabbit anti-mouse secondary antibody (Bioswamp, Item No. PAB160022), the slides were visualized using the chromogenic substrate 3,3′-diaminobenzidine and counterstained with hematoxylin. YB-1 immunopositivity was defined as the presence of any specific staining in the nucleus and or cytoplasm. We calculated the percentage of positive cells, assigning negative a score of 0, ≤10% positive cells a score of 1, 11–50% positive cells a score of 2, 51–75% positive cells a score of 3, and ≥75% positive cells a score of 4. Analysis of immunohistochemical staining of the breast tissue slides was carried out by two pathologists [9].

### 2.3. Cell Culture

MCF-7 and MCF-7/ADR cells were obtained from the Biochemistry Laboratory of Nanchang University (Jiangxi, China). MCF-7 and MCF-7/ADR cells were cultured in 24-well plates in DMEM supplemented with 10% FBS at 37°C with 5% CO_2_ in a humidified environment.

### 2.4. Cell Viability Analysis

Cell viability was measured using the CCK-8 assay (Beyotime, China). Cells were plated at 3 × 10^3^ cells/ml in 96-well plates for 24 h, 48 h, and 72 h, and CCK-8 solution (10 *μ*l) was added to each well. The optical density was measured at 450 nm using a microplate reader (BiTek Instruments, Inc.).

### 2.5. Tumor Spheroid Formation and Cell Morphology

Cells were seeded into 6-well plates at 1 × 10^5^ cells/well and incubated for 15 days at 37°C in a humidified environment containing 5% CO_2_. Repeated pipetting was carried out every five days to separate the clustered cells, and cell morphology was observed using a microscope.

### 2.6. YB-1 Overexpression and Knockdown Vector

pcDNA3.1 HisA was used to construct the YB-1 overexpression vector with DH5*α* as the host bacteria. Total RNAs were isolated from the tissue using an extraction kit, and reverse transcription was accomplished, followed by amplification with polymerase chain reaction (PCR) using forward and reverse primers of YB-1. The reaction procedure was as follows: denaturation at 94°C for 3 min; 30 cycles of 94°C for 45 s, 54°C for 30 s, and 72°C for 60 s; and extension at 72°C for 5 min. The PCR products were collected by gel extraction. Double restriction enzyme digestion by NheI and XhoI was applied to the recovered target gene fragment and eukaryotic expression vector pcDNA3.1 HisA plasmid (Clontech), respectively. The linearized vectors were recycled by a purification column. After the digested PCR gene fragments and linearized vector were mixed at 16°C overnight, the acquired YB-1 overexpression vectors were transfected into competent DH5*α* cells. The PCR primers used were as follows: YB-1 (forward: AGAAGTGATGGAGGGTGCTG and reverse: GCTGTCTTT GGCGAGGAG) and pcDNA3.1 HisA (forward: CTAGAGAACCCACTGCTTAC and reverse: TAGAAGGCACAGTCGAGG).

PCR using (+) and (-) primers was used as the authenticating method for vector construction. The transformant cultivated in the plate was resuspended in 10 *μ*l of lysogeny broth, and 1 *μ*l was used as a template for colony PCR identification. The reaction procedure was as follows: denaturation at 94°C for 5 min; 30 cycles of 94°C for 60 s, 56°C for 60 s, and 72°C for 60 s; and extension at 72°C for 10 min. The PCR product was analyzed by gel electrophoresis.

The YB-1 knockdown vector was constructed with small interfering RNA (siRNA) using a standard procedure involving target gene identification, siRNA designed by GeneChem Co. (GeneChem Co., Ltd., Shanghai, China), siRNA (targeting sequences were 5′-CGGCAATGA AGA AGATAA A-3′) preparation, siRNA transfection, and detection of RNA interference by Western blot and functional study.

The MCF-7/ADR cells were divided into four groups: control, YB-1 overexpression, YB-1 knockdown, and empty YB-1 vector (EV). The vectors were transiently transfected into MCF-7/ADR cells using Lipofectamine 2000 reagent.

### 2.7. Cell Invasion Analysis

To evaluate cell invasion, the upper side of the Transwell membrane was precoated with Matrigel and incubated at 37°C for 30 min to induce gel formation. The membrane was hydrated in phosphate-buffered saline (PBS) for 5 min before use. In the lower chamber, 750 *μ*l of DMEM containing 10% FBS was added, and 1 × 10^5^ cells/well were added to the upper chamber. After 48 h of incubation, the cells were fixed with methanol, stained with crystal violet (0.5% *w*/*v*), and counted. Experiments were performed in duplicates.

### 2.8. Cell Cycle Analysis

Cell cycle progression was evaluated using a cell cycle kit (Bioswamp, China) according to the manufacturer's manual. Cells were cultured in 24-well plates, harvested by centrifugation at 1000 × g for 5 min, and washed twice with ice-cold PBS. Propidium iodide was added and cell cycle progression was evaluated by flow cytometry (Beckman, USA).

### 2.9. Immunofluorescence

Cells were fixed with 4% paraformaldehyde for 30 min, washed three times with PBS for 3 min each, and blocked for 60 min with 5% bovine serum albumin to avoid nonspecific binding. They were then incubated with primary antibodies against YB-1 and BCRP overnight and subsequently with specific secondary antibodies at room temperature overnight. The results were observed using a fluorescence microscope [10].

### 2.10. Quantitative Reverse Transcription PCR (qRT-PCR)

Whole RNA of the tissue and cell samples was extracted using TRIzol reagent according to the manufacturer's procedures, and cDNA was synthesized using a reverse transcription kit (Thermo, USA). qRT-PCR was performed using a real-time system (Bio-Rad) using the SYBR Green PCR Kit (KM4101, Kapa Biosystems). Each reaction was performed in duplicate, and the results were analyzed using the 2^-△△Ct^ method. The primers were designed and configured by Nanjing Kingsy Biotechnology Co., Ltd. The PCR primers used were as follows: YB-1 (forward: AGAAGTGATGGAGGGTG CTG and reverse: GCTGTCTTTGGCGAGGAG), BCRP (forward: GCCYCGGTATTC CATCTT and reverse: GGTTGTTGTAGGGCTCAC), and GAPDH (forward: CAAGTT CAACGGCACAG and reverse: CCAGTAGACTCCACGACAT).

### 2.11. Western Blot

Cell and tissue (50 *μ*g of tissue per ml) samples were treated by an appropriate amount of radioimmunoprecipitation buffer in Eppendorf tubes. The lysates were centrifuged at 12000 × g for 15 min at 4°C. The supernatant was removed and protein concentrations were determined using a commercial bicinchoninic acid kit (Bioswamp, China) according to the manufacturer's instructions. Total protein was separated by 12% sodium dodecyl sulfate-polyacrylamide gel electrophoresis and transferred to polyvinylidene fluoride membranes. The membranes were blocked for 2 h in skim milk, washed five times with Tris-buffered saline/0.1% Tween-20 (TBST), and probed with primary antibodies against YB-1 and glyceraldehyde 3-phosphate dehydrogenase overnight at 4°C. The membranes were then washed three times with TBST and incubated with secondary antibody (horseradish peroxidase-conjugated goat anti-rabbit/mouse, 1 : 1000) for 60 min. Protein expression was evaluated using ImageJ version 1.38 (Tianneng, Ltd., Shanghai) [11].

### 2.12. Tumorigenicity in BALB/c Nude Mice

BALB/c mice (4–6 weeks old, females, 200–220 g) purchased from Beijing Huafukang Bioscience Co., Inc. were used for the in vivo experiments. The mice were given free water and commercial feed pellets and maintained at 24°C in a 12/12 h light/dark cycle. The subcutaneously implanted tumor model was constructed in mice using MCF-7 or ADR-resistant MCF-7 (MCF-7/ADR) cells. The mice were divided into four groups: MCF-7/ADR, MCF-7/ADR+ADR, MCF-7, and MCF-7+ADR. The mice were administered sterile saline (MCF-7 and MCF-7/ADR groups) or ADR (4 mg/kg) (MCF-7/ADR+ADR and MCF-7+ADR groups) for two weeks. The sizes and weights of tumors were measured after two weeks. The tumor volume was calculated by the following equation: (length^∗^ width^2^)/2 = Volume (mm^3^) [5], and tumor tissue was removed for cell culture and pathological examination. Cells obtained from the tumor tissue were cultured in 24-well plates in Dulbecco's modified Eagle medium (DMEM) supplemented with 10% fetal bovine serum (FBS) at 37°C with 5% CO_2_.

### 2.13. Statistical Analysis

Clinical data were analyzed using SPSS19.0 software. Data and statistical analysis of both the in vivo and in vitro experiments were performed using OriginPro (OriginLab, Northampton, USA) and GraphPad Prism (GraphPad Software, San Diego, USA) software. Data are presented as the mean ± standard error of the mean.

## 3. Results

### 3.1. The Expression Level of YB-1 Is Related to the Clinicopathological Characteristics

**Figure 1:**
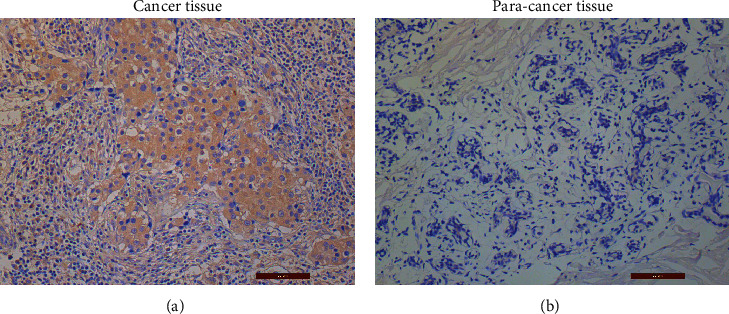
Immunohistochemical staining of breast cancer tissue (a). Immunohistochemical staining of breast paracancer tissue (b) (magnification 200x).

The expression of YB-1 was found in both cancer and paracancerous tissues, but in paracancerous tissues, nuclear staining was not found. Nuclear staining was common in cancer tissues (Figure 1(a)). According to the positive value of immunohistochemical staining, the expression level of YB-1 in cancer tissues is significantly higher than that in paracancerous tissues (Figures 1(a) and 1(b)). In the whole cohort (Table 1), comparing the YB-1-positive value of immunohistochemical staining to relevant clinicopathological characteristics, patients with positive lymph nodes had a significantly higher value of YB-1 staining than patients with negative lymph nodes (*P* = 0.017). Therefore, the high YB-1 staining value is related to lymph node metastasis. Despite not being significantly different, patients with a value of 3–4 tended to be slightly younger, mostly focused in the ≤35-year-old group and 35–49-year-old group. There were no significant differences in tumor size, histological grade, subtype, and pathological stage between two groups (Table 1).

### 3.2. Successful Preparation of MCF-7/ADR Cells and the Characteristics in MCF-7 and MCF-7/ADR

**Figure 2:**
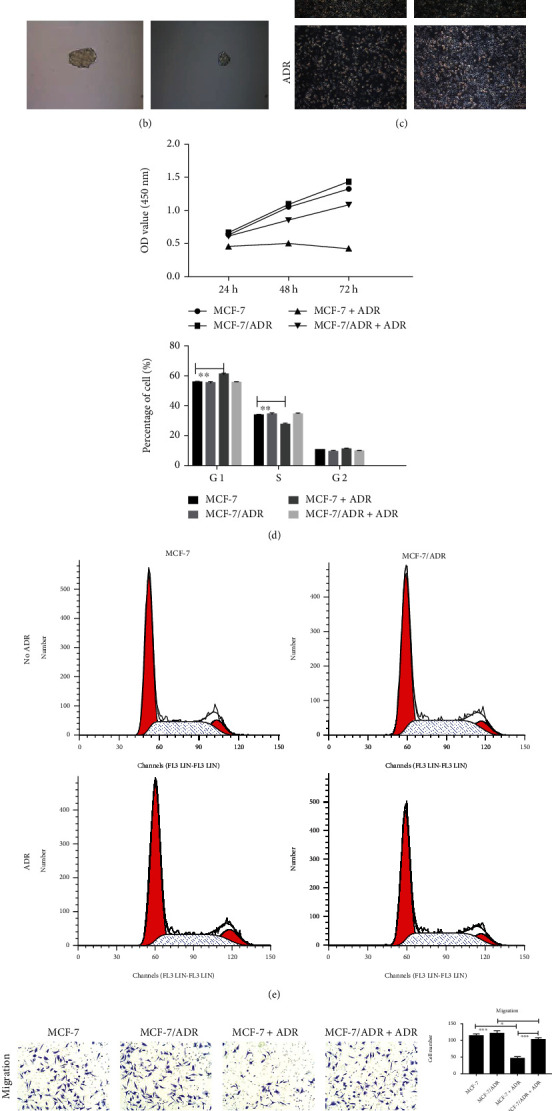
Proliferation, cell cycle progression, invasion, and migration were evaluated in MCF-7/ADR cells. (a) The ADR concentration used to establish the ADR-resistant cell line was determined by MTT assay and resistance of MCF-7/ADR to other drugs (CDDP and 5-FU). (b) Tumor spheroid formation (magnification 40x) and (c) cell morphology (magnification 100x). (d) Cell proliferation was detected by CCK-8 in 24 h, 48 h, and 72 h. (e) Cell cycle progression was examined by flow cytometry. (f) Cell invasion and migration were assayed by Transwell test (magnification 200x). Value was presented for three times to conduct statistical analysis. (g) The weight and size (long and short diameter) of tumors formed in the mice. (h) Comparison of YB-1, BCRP, cyclin E, cyclin D1, p53, and GAPDH expressions in different groups by Western blot. Value was presented for three times to conduct statistical analysis. In vivo experiments *N* = 6, in vitro experiment *N* = 3, ^∗^ means *P* < 0.05, ^∗∗^ means *P* < 0.01, and ^∗∗∗^ means *P* < 0.001.

According to Figure 2(a), the IC50 of ADR was 200 ng/ml in the normal MCF-7 cell line, and this concentration was applied to establish the ADR-resistant cell line. MCF-7/ADR was resistant not only to ADR but also to cisplatin (CDDP) and fluorouracil (5-FU). MCF-7 cell tumorigenesis tests showed that MCF-7 had strong tumorigenic ability (Figure 2(b)). MCF-7 cells formed tumor clusters that grew and adhered tightly, whereas the MCF-7/ADR cells grew loosely with poor intercellular adhesion (Figure 2(c)). In addition, when treated with ADR, MCF-7/ADR cells exhibited increased proliferation (Figure 2(d)), migration, and invasion (Figure 2(f)) compared to those of MCF-7 cells, but there was no significant difference in cell cycle progression (Figure 2(e)).

### 3.3. YB-1 Is Overexpressed in Mice Implanted with MCF/ADR

In the vivo experiments, in terms of tumor growth in mice, the tumor volume induced by MCF-7/ADR was significantly larger than those induced by MCF-7, whether with or without ADR treatment (Figure 2(g)). We found that the protein expressions (YB-1, BCRP, cyclin E, cyclin D1, and P53) of mice tissue implanted with MCF/ADR cells were higher than that of MCF cells, whether with or without ADR treatment. The differences in protein expression between the two ADR treatment groups were more significant (Figure 2(h)).

### 3.4. The Effect of YB-1 on the Proliferation, Cell Cycle Progression, and Migration of MCF-7 and MCF-7/ADR Cells

**Figure 3:**
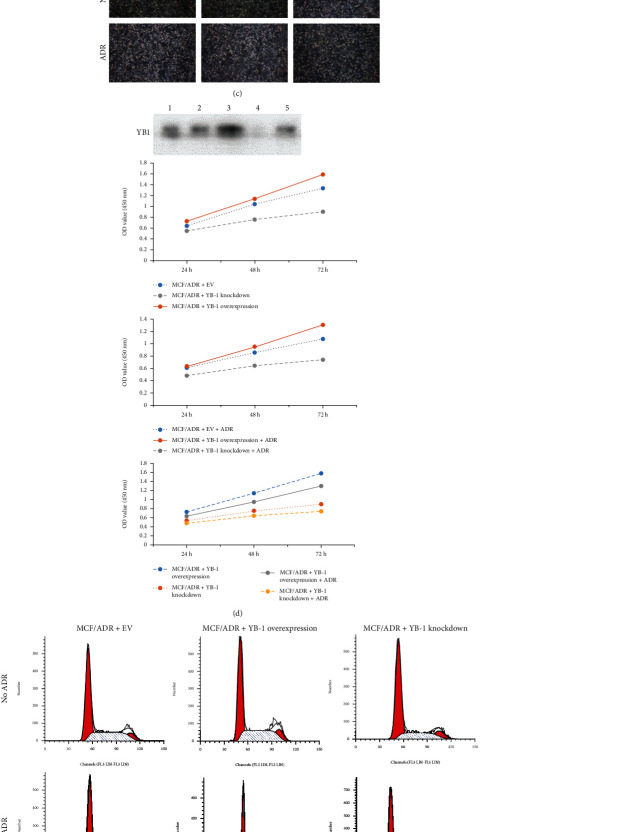
The effect of YB-1 on cell morphology, proliferation, and cell cycle in MCF-7 and MCF-7/ADR cells. (a) The expression of YB-1 in four group cells. (b) The expression of YB-1 in MCF-7 and MCF-7/ADR cells treated with YB-1 overexpression and knockdown. 1: MCF-7; 2: MCF-7/ADR; 3: MCF-7/ADR+YB-1 overexpression; 4: MCF-7/ADR+YB-1 knockdown; 5: MCF-7/ADR+YB-1 EV. (c) Cell morphology was observed by optical microscopy (magnification 100x). (d) Cell proliferation was detected by CCK-8. (e) Cell progression was examined by flow cytometry. Value was presented for three times to conduct statistical analysis. *N* = 3, ^∗^ means *P* < 0.05, ^∗∗^ means *P* < 0.01, and ^∗∗∗^ means *P* < 0.001.

In vitro experiments, the resistance of drug-resistant MCF-7/ADR cells may be related to the expression of YB-1. The expression of YB-1 mRNA was significantly higher than that of MCF-7 cells in MCF-7/ADR cells, and ADR treatment markedly decreased the expression of YB-1 mRNA in MCF-7 cells. However, the expression of YB-1 mRNA was not significantly decreased in drug-resistant cell lines (Figure 3(a)). Moreover, the expression of YB-1 protein was similar to the expression of YB-1 mRNA.

**Figure 4:**
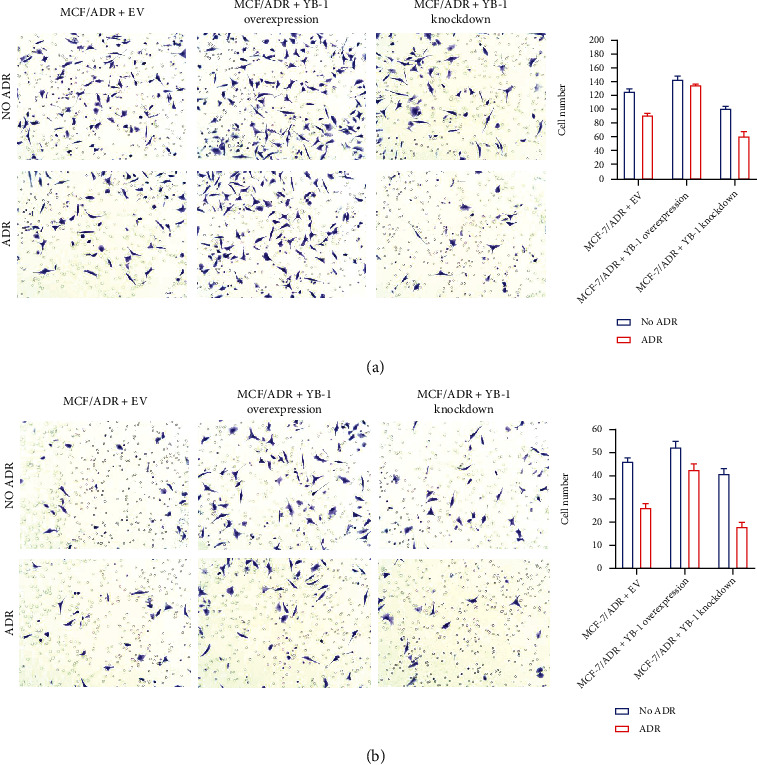
The effect of YB-1 on migration and invasion in MCF-7 and MCF-7/ADR cells. (a) Cell migration was assayed by Transwell test (magnification 200x). (b) Cell invasion was assayed by Transwell test (magnification 200x).Value was presented for three times to conduct statistical analysis. *N* = 3, ^∗^ means *P* < 0.05, ^∗∗^ means *P* < 0.01, and ^∗∗∗^ means *P* < 0.001.

To explore the effect of YB-1 on MCF-7 and MCF-7/ADR cells, YB-1 overexpression and knockdown vectors were successfully constructed (Figure 3(b)). In addition, compared with control cells, those subjected to YB-1 overexpression were in a better state, showing more compact adhesion. However, YB-1 knockdown rendered the cells in poor condition, including intercellular adhesion and individual dissociation. With ADR treatment, the same trends were observed in all cells (Figure 3(c)). Cell proliferation was promoted by YB-1 overexpression and restricted by YB-1 knockdown, and ADR treatment decreased proliferation in all cells (Figure 3(d)). The cell cycle was also altered by YB-1 overexpression/knockdown. Compared with MCF-7/ADR+YB-1 EV, YB-1 knockdown increased the number of cells in the G1 phase, whether with or without ADR treatment (Figure 3(e)). Furthermore, cell migration and invasion were increased by YB-1 overexpression and decreased by YB-1 knockdown. ADR treatment did not affect the migration and invasion of cells subjected to YB-1 overexpression, but in cells subjected to YB-1 knockdown, migration and invasion were decreased (Figures 4(a) and 4(b)).

## 4. Discussion

In our study, we retrospectively analyzed the relationship between the clinicopathological data of 48 breast cancer patients and the expression of YB-1 in both breast cancer tissue and paracancerous tissue specimens by immunohistochemical staining. According to the immunohistochemical study interpretation standards and the judgment of two pathologists, YB-1 expression was divided into scores of 0, 1, 2, 3, and 4. The results revealed that the expression of YB-1 in paracancerous tissue was mostly scored as 0–1 and nuclear staining was not found, while in cancer tissue with scores of 2–4, nuclear staining was common. This was consistent with most findings in the literature [12, 13]. Combined with clinicopathological data, the expression of YB-1 in patients with regional lymph node metastasis had significant differences. In other words, patients with high expression of YB-1 were prone to regional lymph node metastasis. Although there was no significant difference in age, higher expression in patients younger than 49 years old was found, especially in patients younger than 35 years old. Seven patients had YB-1 expression scores of 3–4, accounting for 63.6% of patients.

YB-1 was an indicator of poor prognosis in many tumors [14]. YB-1 is associated with intestinal-type gastric cancer lymph node metastasis [15]. In our cohort, higher YB-1 expression correlated with regional lymph node metastasis and a tendency toward younger age. Age and lymph node metastasis were also poor prognostic factors. Therefore, YB-1 could be used as one of the prognostic factors in breast cancer.

Previous researchers [5] analyzed prognostic factors of recurrence by detecting plasma YB-1 levels. However, the detection of YB-1 in tissues using immunochemical-analyzed prognostic factors has been rare. This method is more practical and economical than measuring plasma YB-1 levels.

In vivo experiments, YB-1 expression was increased in MCF-7/ADR cells, with or without adriamycin treatment. To further explore the effect of YB-1 expression on the effect of adriamycin, in this study, pcDNA3.1 HisA and siRNA were used to construct overexpression vectors and knockdown vectors, respectively. In the in vitro experiments, the YB-1 overexpression cell lines had greater cell proliferation, migration, and invasion than the YB-1 knockdown and EV cell lines. ADR treatment did not affect the migration and invasion of cells subjected to YB-1 overexpression, but it decreased the migration and invasion of cells subjected to YB-1 knockdown. Therefore, overexpression of YB-1 can increase cell resistance to adriamycin, and YB-1 can also be used as one of the predictors of resistance to adriamycin.

YB-1 is a transcription and translation factor [16], and the main mechanisms of drug resistance are drug efflux and gene regulation disorders [17, 18]. This will affect not only the efficacy of chemotherapy but also the efficacy of endocrine therapy, with overexpression of YB-1 degrading the expression of ERs, leading to endocrine resistance [19]. YB-1 phosphorylation and nuclear translocation are the keys driving drug resistance in tumor cells. Phosphorylation of the YB-1 cold shock domain at Ser102 is a necessary condition for the nuclear translocation of YB-1 in tumor cells [20]. Its upstream enzymes and signaling pathways, such as Akt and the Akt/mTOR, and MEK/ERK signaling pathways activate and regulate YB-1 nuclear translocation [21, 22]. PI3K, Mtorc1, and RSK inhibitors were also found to inhibit Ser102 phosphorylation, YB-1 phosphorylation, and nuclear translocation [23]. Targeting the AKT/mTOR/p70S6k and/or MEK/ERK/p90RSK signaling pathway to inhibit downstream effector YB-1 activity is expected to be a new antitumor therapy to overcome drug resistance [7].

This study presents several limitations. Due to the short follow-up time, the recurrence and survival rates of the patients could not be analyzed. Prolonging the follow-up time, this part of the data will be supplemented in the future. In clinical specimen processing, if PCR is used for quantitative analysis, human subjective factors can be discarded to make the data more accurate and convincing. Further, as translation factor, the target genes should be identified in the future. The affected pathways should also be identified. The follow-up time of the patients should also be prolonged.

## 5. Conclusions

Our study provides the evidence that the expression of YB-1 in tumor tissues is associated with regional lymph node metastasis, and the patients with high expression of YB-1 are younger. The proliferation, migration, and invasion of YB-1 overexpressed tumor cells were enhanced, and they were resistant to adriamycin. This study is a small sample study, and it will expand the samples and perfect the follow-up data in the future to further supplement the deficiencies.

## Figures and Tables

**Table 1:** Clinical and pathological characteristics of the patients.

	Number	*P* value
Number of patients	48	
Gender		
Female	48	
Median age (range) (years)	46 (27-70)	
Age (years)		0.057
≤35	11 (22.9%)	
35-49	20 (41.6%)	
50-70	17 (35.4%)	
Tumor size (diameter, cm)		
<5 cm	28 (58.3%)	0.314
≥5 cm	20 (41.7%)	
Histological grade (*n* (%))		0.234
Grade 2	21 (43.8%)	
Grade 3	27 (56.2%)	
Lymph node status (*n* (%))		0.017
Negative	21 (43.8%)	
Positive	27 (56.2%)	
Subtype (*n* (%))		0.320
Luminal A	6 (12.5%)	
Luminal B	21 (43.8%)	
Triple negative	10 (20.8%)	
HR-, Her2+	11 (22.9%)	
Pathological stage at diagnosis (*n* (%))		0.229
Stage II	28 (58.3%)	
Stage III	20 (41.7%)	

## Data Availability

All data for this article are available from the corresponding author.
